# Extracts from the Minutes of the Atlanta Academy of Medicine

**Published:** 1871-04

**Authors:** 


### EXTRACTS FROM THE MINUTES OF ATLANTA ACADEMY OF MEDICINE.

February 3d, 1871.

Reports of cases being in order.

Dr. J. H. Lowe reported what he termed a case of prolapsus uteri, in a subject forty-three years old. Complains of burning sensation in the feet frequently, which is relieved to some extent by pediluvium of solution of common salt. Ulcers were found on the os uteri, and greatly benefitted by the application of Arg. Nit. Elongated cervix with contracted canal were found to exist. He desired the opinion of members as to the best mode of treatment in order to give permanent relief from the various distressing nervous symptoms attending such cases.

Dr. J. G. Westmoreland suggested the propriety and importance of further investigation, with the view of determining whether or not chronic inflammation exists in the intra-uterine mucous membrane.

Dr. W. F. Westmoreland, by request of Dr. Lowe, gave his opinion of the best manner of dilating the cervical canal. He is in the habit of using the seat-angle, and thinks it preferable, in many respects, to the sponge tent. Considers dilatation a pre-requisite in most cases, to the local treatment of endo-metritis.

Dr. Logan, President of the Academy, whose opinion of the nature and treatment of such affections was desired, expressed the opinion that in the case reported, endo-metritis is the probable difficulty to be treated. Prolapsus, under such circumstances, sometimes, though rarely, requires attention by way of giving artificial support. Recently, a case received great relief from the use of a uterine supporter. Dilatation is generally necessary previous to the application of remedies to the diseased surface in cases of endo-metritis. His mode of introducing agents into the uterus is that of carrying by a probe bits of cotton saturated with the solution of carbolic acid, nitrate of silver, or whatever article be used, to the affected part, and leaving them in contact with the surface. Owing to the unpleasant effects which sometimes result from the injection of liquid preparations into the uterus, he has abandoned the practice.

Dr. J. G. Westmoreland advocated the cautious use of uterine injections with the graduated hard-rubber uterine syringe in treatment of chronic endo-metritis. Uses only eight or ten drops of catheretic solutions at each insertion of the nozzle, repeating, at the same time, as often as circumstances seem to demand, but discharging this small quantity at different points in the cavity. Failure of success in the treatment of this disease no doubt often arises from neglecting to relieve the engorged condition of the uterus at the outset, by leeching or scarification.

Dr. Withers' experience in the treatment of endo-metritis satisfies him that the introduction by a probe lint saturated with a solution of nitrate of silver, after the cervical canal is properly dilated, is the proper mode of treatment.

Dr. Love gave notice that he had in process of preparation a written report of some cases of cerebro-spinal meningitis, treated by Dr. Ingraham and himself, and that it will be read at next regular meeting. He referred to the subject of uterine disease—in the investigation of which the meeting had been engaged during the evening—by saying he had for several years, applied remedies to the intra-uterine mucous membrane by means of an instrument of his own invention. With this sponge is introduced into the cavity, and saturated with liquid preparations afterwards, through a tube, to the end of which it is attached. He thinks spasm of the womb, or "uterine colic," as it is called, is the result of temperature and chemical action of the liquids injected.

---

FEBRUARY 10th, 1871.

DR. LOVE, in accordance with notice at last meeting, presented a written report on cases of cerebro-spinal meningitis, in which he presented some facts of special interest, in a physiological point. In one case, that of a child aged 11 years, no *diagnostic* symptom of *meningitis* presented itself until within two hours of the time when respiration ceased. The heart's action continuing, irregular, for a minute or two after the breathing had stopped. Artificial respiration was resorted to, under which the heart's action became regular, with pulse 112 at the end of an hour. At the end of two hours, pulse 120; and at the end of *two hours and fifty-three minutes, from cessation of respiration*, cardiac action ceased,—the air passages having filled with fluids to such a degree that the lungs could no longer be artificially inflated. The cessation of the normal respiration seemed to depend solely upon a suspension of the *exceto-motor powers of the respiratory nerves;* and no efforts nor applications would arouse them to action in the least degree. Under the artificial respiration, capillary circulation returned, together with the warmth, glow, and secretion about the surface; the blood arterialized regularly, and every indication of normal physiological action presented, save the suspension of action in the muscles of respiration. Dr. Love gave it as his opinion that these phenomena *located* the *lesions* or *effusions*, in this case, *at the origin, or along the line of the phrenic and inter-costal nerve;* the effusions, however limited, resulting in paralysis—the paralysis proving fatal.

On the question of the pathology of cerebro-spinal meningitis, Dr. Love takes the position that it is more nearly allied to erysipelas and diphtheria than to any other special disease to which it could be likened—a disease attacking the serous membranes—having a tendency to metastate from one to another, and to terminate in effusions or exudation; thus leading to fatal results. Through this tendency to metastasi, Dr. Love explains the protean character of the disease, and accounts for the varied and changing symptoms presented in different cases, or in the same case, at different stages.

He suggests as salient points of attack in the treatment, to divert the disease (taking advantage of its tendency to metastate) by endeavoring to centre its force on the less vital parts, organs, or tissues, and the neutralization of the poisonous influences in the system, by the use of such remedies as have been found most effective in the treatment of those more nearly allied diseases—erysipelas and diphtheria—the three forms having a kindred pathology: erysipelas attacking the skin and subculaneous tissues, diphtheria the mucous membranes, while this spends its force on the serous and nervous.

Dr. Logan desired information of members as to the prevalence of cerebro-spinal meningitis in the city at this time as an epidemic. He thought the limited number of cases which had recently occurred in town would not authorize such an opinion.

Drs. Moore, O'Keefe, Ray, Love, Boring, and Westmorelands, responded in a few remarks; a majority of whom expressed the belief that as a distinct epidemic the disease does not prevail; that the influence existing here for several months, and manifested in the general epidemic of influenza, is identical with that which produces the sporadic cases of meningitis recently met with in town.

